# Pilot Exploratory Study of Serum Differential Scanning Calorimetry in Aneurysmal Subarachnoid Hemorrhage Reveals Preliminary Outcome-Related Proteome-Level Thermodynamic Patterns

**DOI:** 10.3390/jcm15031139

**Published:** 2026-02-02

**Authors:** Dénes Lőrinczy, Peter Csecsei

**Affiliations:** 1 Department of Biophysics, Medical School, University of Pécs, Szigeti Str. 12, 7624 Pecs, Hungary; denes.lorinczy@aok.pte.hu; 2 Department of Neurosurgery, Medical School, University of Pécs, Rét Str. 2, 7624 Pecs, Hungary

**Keywords:** differential scanning calorimetry, thermal liquid biopsy (TLB), aneurysmal subarachnoid hemorrhage, clinical outcome

## Abstract

**Background:** Aneurysmal subarachnoid hemorrhage (aSAH) induces complex systemic inflammatory and metabolic responses that may influence clinical outcome. DSC provides an integrative biophysical readout of proteome-level thermodynamic behavior rather than protein-specific identification or quantification; however, its applicability in neurocritical conditions remains largely unexplored. This pilot study aimed to explore whether serum DSC profiles show preliminary associations with clinical severity and neurological outcomes after aSAH. **Methods**: Serum samples collected on day 1 after aSAH were analyzed by DSC and compared with healthy control samples. A small patient cohort was stratified according to clinical severity and neurological outcome. Thermograms were evaluated based on melting temperatures (Tm), calorimetric enthalpy (ΔHcal), heat capacity changes (ΔCp), and the relative contributions of major serum protein components. **Results:** Healthy controls exhibited characteristic DSC profiles dominated by a cooperative albumin transition at approximately 65–66 °C. In this limited cohort, patients with severe clinical conditions and unfavorable outcomes displayed marked thermogram reorganization, including increased albumin Tm, reduced unfolding cooperativity, decreased ΔCp, and enhanced high-temperature immunoglobulin-related contributions. Patients with mild condition and favorable outcome showed profiles more similar to those of the controls. Notably, patients with severe conditions but favorable outcomes demonstrated heterogeneous albumin-related thermal domains, which may reflect individual-level variability and suggesting dynamic proteomic heterogeneity at the early post-ictus phase. Given the small group sizes, these patterns should be interpreted as exploratory and hypothesis-generating. **Conclusions**: This pilot exploratory study suggests that serum DSC may capture preliminary thermoanalytical patterns associated with clinical outcomes after aSAH. While the findings indicate the potential of DSC as a systems-level tool in neurocritical care, larger, well-powered studies are required to validate these observations and assess their robustness and generalizability.

## 1. Introduction

Aneurysmal subarachnoid hemorrhage (aSAH) is a critical neurological condition characterized by bleeding into the subarachnoid space due to the rupture of an intracranial aneurysm. This event accounts for approximately 85% of nontraumatic subarachnoid hemorrhages and poses significant morbidity and mortality risks. The global incidence of aSAH is estimated at 7.9 per 100,000 person/year, with Japan and Finland showing higher prevalences [1]. The pathophysiology of aSAH involves several complex mechanisms. Hemodynamic stress is a primary factor in the formation of intracranial aneurysms, particularly at arterial bifurcations where turbulent blood flow can weaken vessel walls [2]. Following aneurysm rupture, blood enters the subarachnoid space, leading to increased intracranial pressure and potential cerebral ischemia. Complications such as cerebral vasospasm can occur days after the initial hemorrhage, peaking between the fifth and seventh days, which contribute to delayed cerebral ischemia and neurological deficits [3]. Current management strategies for aSAH focus on early aneurysm repair to prevent rebleeding, typically through surgical clipping or endovascular coiling. Supportive care includes maintaining euvolemia, controlling intracranial pressure, and administering calcium channel blockers like nimodipine to mitigate cerebral vasospasm [4,5]. Despite these interventions, patient outcomes remain variable, highlighting the need for improved diagnostic and therapeutic approaches.

Differential scanning calorimetry (DSC) analysis of different body fluids (recently termed thermal liquid biopsy (TLB) [6,7,8,9]), which assesses heat changes associated with biochemical reactions, has emerged as a potential tool for evaluating metabolic alterations in various medical conditions as it provides complementary information to aid in diagnosis [10,11,12,13,14,15,16,17,18,19,20,21]. Importantly, DSC does not resolve individual proteins or provide molecular proteomic identification. Instead, it captures the ensemble thermodynamic behavior of the circulating serum proteome, which is shaped by the protein composition, post-translational modifications, ligand binding, and protein–protein interactions. In the context of aSAH, these measurements could provide insights into the metabolic state of patients by detecting subtle changes in serum thermogenic properties. Such data may reflect underlying inflammatory responses, energy metabolism disruptions, or other pathophysiological processes following hemorrhage. For instance, studies have noted correlations between body temperature fluctuations and cerebral energy metabolism in aSAH patients, suggesting that thermal measurements could serve as indirect markers of metabolic activity and injury severity [22].

Our first attempt to apply calorimetric techniques on serum samples from aSAH patients holds promise for enhancing our understanding of disease mechanisms and potentially guiding therapeutic interventions. By monitoring metabolic changes, clinicians may be better equipped to predict patient outcomes, tailor treatments, and improve overall management strategies for this life-threatening condition.

## 2. Materials and Methods

### 2.1. Study Design and Population

Institutional review board approval was obtained previously (IV/8468-1/2021/EKU). Written informed consent was obtained from patients or their legal representatives prior to their inclusion in the study. Patients diagnosed with aneurysmal subarachnoid hemorrhage (aSAH) at our institution from February 2021 to November 2023 were prospectively included in the study. Our study’s inclusion criteria were as follows: 1. age > 18 years old; 2. confirmed diagnosis of aSAH by noncontrast head computer tomography (CT) and established diagnosis of aneurysm by CT angiography (CTA) or digital subtraction angiography (DSA); and 3. diagnosis occurred within 24 h after the index event. Patients with traumatic SAH, were pregnant, were admitted to the hospital more than 24 h after the ictus, had no aneurysm treatment, had bleeding from arteriovenous malformation, did not sign a consent form, had underlying systemic diseases (malignancies, liver and/or renal insufficiency, chronic lung disease, inflammatory bowel disease, or any known chronic gastrointestinal diseases), or had a chronic infection or signs of any acute infection on admission were excluded. Patients who experienced re-rupture after the ictus or deteriorated during treatment were also excluded from the study. For eligible patients, their medical records, including age, sex, risk factors (hypertension, diabetes, and smoking status), blood pressure and laboratory measurements on admission, baseline World Federation of Neurological Societies (WFNS) score and modified Fisher (mFisher) score, interventions performed during intensive care (mechanical ventilation, extraventricular drain, lumbar drain, etc.), occurrence and type of infection, occurrence of delayed cerebral ischemia (DCI), and clinical outcomes (modified Rankin Scale, mRS), were reviewed and recorded. All patients were followed up through phone calls or personal visits. The mRS score at 3 months was recorded to assess the clinical outcome. A favorable outcome was defined as an mRS score of 0–3 at the follow-up [23]. Clinical severity on admission was assessed using the World Federation of Neurological Societies (WFNS) grading system, as originally defined by Vergouwen MD et al. [24]. The outcome data were assessed by independent, trained, blinded professionals who were not associated with the study at 3 months (±5 days) after the aSAH event. All patients received nimodipine 6 times 60 mg per os from the first day for vasospasm prevention. Delayed cerebral ischemia (DCI) was identified based on previously established criteria [25]. The diagnosis of DCI was made only after thoroughly ruling out other potential causes and required the consensus of at least two neurointensivists. We considered a patient to be DCI-positive if clinical deterioration and/or ischemic lesions (confirmed by CT/MR) occurred within 6 weeks of admission and could not be explained by other causes. Large vessel vasospasm, in the absence of clinical symptoms or a new ischemic lesion, was not considered as DCI [25]. The criteria for defining systemic and central nervous system infection included the presence of infection symptoms with fever (>38 °C), elevated levels of C-reactive protein (CRP) (level continues rising after an initial peak), and/or procalcitonin > 0.5 ng/mL, combined with a positive result from a diagnostic test, such as a chest X-ray or cerebrospinal fluid (CSF), blood culture, or urine analysis. In these patients, we collected serum samples and confirmed that they did not meet any exclusion criteria. Ongoing infections, which could affect the measured marker levels, were ruled out through laboratory tests.

### 2.2. Sampling and Analysis of Investigated Body Fluids

Arterial blood samples were collected from all eligible patients on the 1st day (D1) after ictus. These samples were centrifuged within 30 min (rcf = 4000× *g* at temperature 4 °C), and the serum supernatant was stored at −80 °C until analysis.

### 2.3. DSC Measurements

The thermal unfolding of the human serum was monitored using a SETARAM Micro DSC-III calorimeter (KEP Technologies Group, Caluire-et-Cuire, France), as previously described for plasma and serum samples [26,27,28,29,30]. Our DSC protocol differs from other protocols because our equipment is a heat-flow and not a power-compensated instrument. The maximum volume of our hermetically closed sample holder is 1 mL, while other instruments typically use spiral capillary holders. We used the original physiological sample concentration, whereas other protocols often apply 5–25-fold dilutions. The reason for performing dilutions in capillary-based systems is to reduce aggregation at higher temperatures, which can complicate cleaning and cause deformation of the capillaries. Monaselidze et al. [31] demonstrated that the denaturation behavior of diluted plasma can significantly differ from that of plasma at physiological concentrations, indicating that such data should be interpreted with caution. Due to the larger sample volume used in our setup, the effective heat capacity of the sample is higher; therefore, at elevated heating rates, a substantial temperature lag may occur between the programmed and actual sample temperature. Consequently, an optimal compromise between heating kinetics and allowing the sample to follow the temperature program must be achieved. Based on our own experience and previous reports, a heating rate of 0.3 K·min^−1^ was selected as a suitable balance between sensitivity and thermal equilibrium, allowing for the detection of subtle structural changes in serum proteins at the expense of longer measurement times. All experiments were conducted between 0 and 100 °C. The heating rate was 0.3 K·min^−1^ in all cases. Conventional Hastelloy batch vessels were used during the denaturation experiments, which have an average sample volume of 950 μL. Reference measurements were performed using physiological saline (0.9% NaCl), which was selected to provide ionic strength and osmotic conditions comparable to native serum while lacking thermally active protein components. The sample and reference holders were equilibrated with a precision of ±0.1 mg and prior to each scan, both cells were allowed to equilibrate at the starting temperature for at least 15 min to ensure thermal stability. The repeated scan of the denatured sample was used for baseline correction and was subtracted from the original DSC curve. Heat flow was plotted as a function of temperature. During the evaluation of thermal parameters, the denaturation (melting) temperature (Tm, defined as the temperature at the maximum heat flow) and the calorimetric enthalpy (ΔHcal) were calculated from the area under the heat flow curve in the 40–90 °C range using two-point SETARAM peak integration. Thermal data are presented as the mean ± standard deviation, rounded to one decimal place for temperature values and two decimal places for calorimetric enthalpy. All serum samples were stored at −80 °C until analysis and were subjected to a single freeze–thaw cycle; repeated freeze–thawing was avoided to minimize artefactual protein denaturation. Visibly hemolyzed samples, identified by visual inspection after centrifugation, were excluded from the DSC analysis.

## 3. Results

### DSC Measurements

Samples from healthy control subjects were included and analyzed in parallel with patient samples to provide age-appropriate reference DSC profiles. The controls were selected so that their age distribution was similar to that of the aneurysmal subarachnoid hemorrhage cohort. They consisted of adult volunteers aged 45–70 years, which is comparable to the age range of the patient group (mean age: 55.8 ± 14 years), thereby minimizing age-related effects on serum protein composition and thermal behavior. The inclusion criteria for the controls were the absence of acute or chronic inflammatory disease, malignancy, cerebrovascular disease, recent infection or surgery, and no regular medication known to affect serum protein binding. The blood sampling, serum handling, storage, and DSC measurement conditions were identical to those used for patient samples. Given the exploratory pilot design and consecutive enrollment of all eligible participants according to predefined criteria, no formal flow diagram was generated.

**Figure 1:**
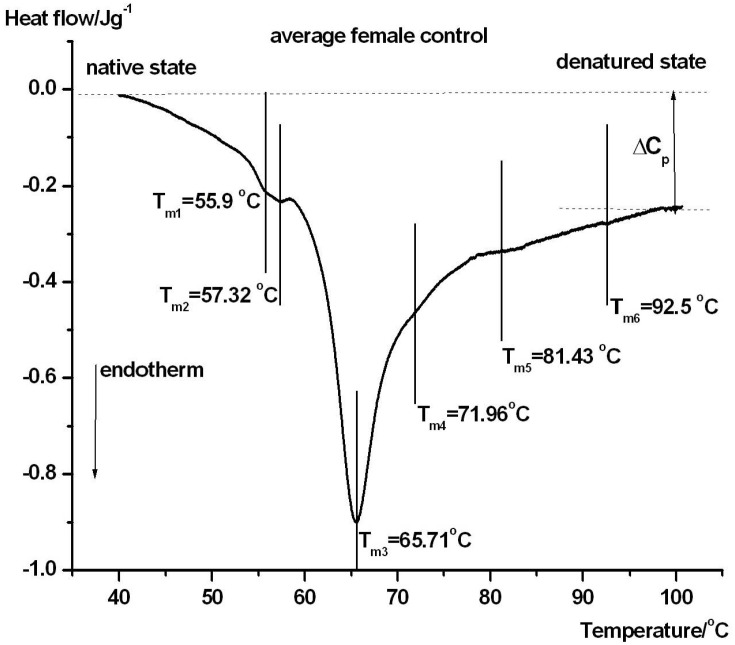
DSC scan of a female healthy control’s blood serum. *T*_*m*1–6_ indicates the denaturation peak of a possible thermal domain. The endotherm deflects downward. Δ*C_P_* is the heat capacity increase after denaturation compared to the native state. The slope of the DSC curve reflects the temperature-dependent changes in heat capacity during protein unfolding, while peak maxima indicate denaturation (melting) temperatures (Tm) and the area under each peak corresponds to the associated calorimetric enthalpy contribution (ΔHcal).

**Figure 2:**
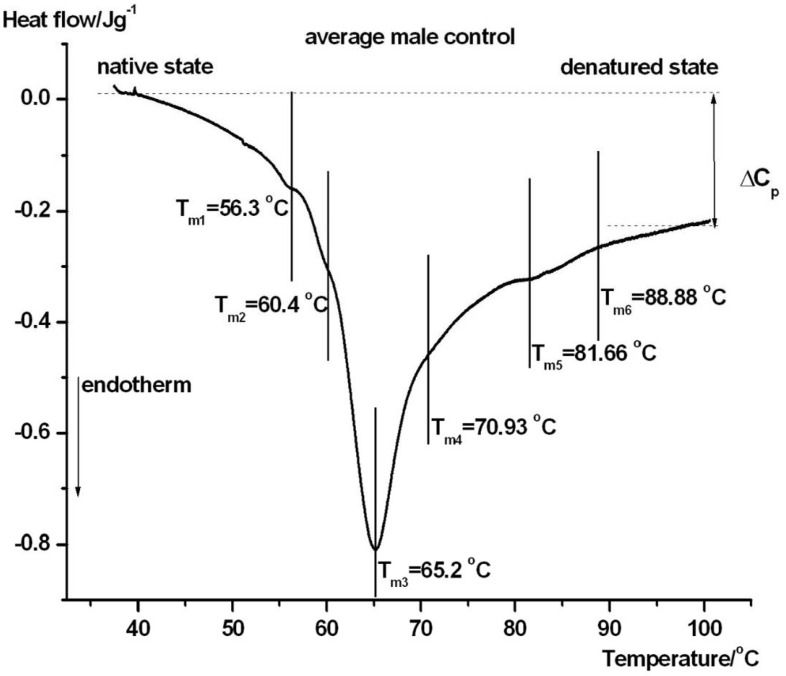
DSC scans of a male healthy control’s blood serum. *T*_*m*1–6_ indicates the denaturation peak of a possible thermal domain. The slope of the DSC curve reflects the temperature-dependent changes in heat capacity during protein unfolding, while peak maxima indicate denaturation (melting) temperatures (Tm) and the area under each peak corresponds to the associated calorimetric enthalpy contribution (ΔHcal). The symbols are the same as those in Figure 1.

Figure 1 and Figure 2 show the denaturation characteristics of plasma from a healthy female (#5) and male (#5) control patient.

The main transition temperature (when denaturation of albumin, which is main compound in serum, occurs) was nearly the same in both groups (65.7 and 65.2 °C). Based on this and despite the observation that there were mild differences in the other components of *T_m_*, we did not examine gender differences because of the small number of patients and only focused on differences based on the severity of the symptoms.

**Figure 3:**
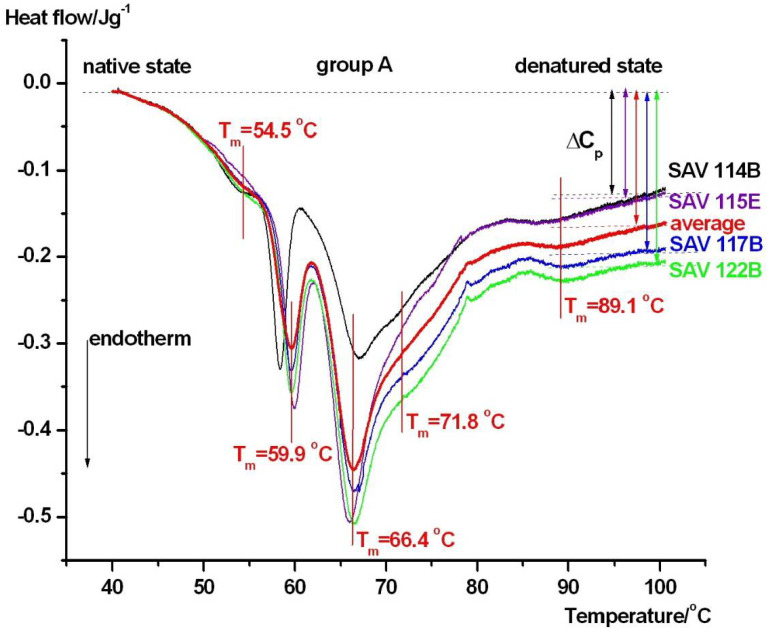
Thermal denaturation curves of patients in group A, who were in a severe clinical state and had poor outcomes. The denaturation peaks of possible thermal domains are marked on the average curve (red).

A total of eight patients were included in the thermoanalytical study. Samples were collected from these patients within 24 h following the ictus. Fifty percent of these patients were female (*n* = 4). The mean age of the entire cohort was 55.8 ± 14 years (mean ± SD). The admission WFNS score was 5 (IQR: 3.5–5), while the 3-month mRS score was 4.5 (2–5.22). The admission creatinine level was 64 (47–76) mmol/L. Seven out of eight patients received an external ventricular drain (EVD) and required mechanical ventilation, and three patients also received a lumbar drain during hospitalization. Using the WFNS and mRS scores, the patients were divided into three groups. Patients in **group A** had a median WFNS score of 5, median mRS score of 5.5, severe clinical state, and poor outcome. Their DSC scans can be seen in Figure 3.

It is widely accepted in the literature [20,21] that denaturation peaks of blood plasma/serum samples can be assigned to the following compounds: fibrinogen at ~51 °C (cannot be detected in serum because it transforms into fibrin), HSA (albumin) and haptoglobulin at ~62 °C, IgG and IgA (globulins) at ~71 °C, and IgG and transferrin at ~82 °C. Comparing the DSC scans of group A and the controls, there is an obvious increase in denaturation temperature and decreased enthalpy contribution of albumin around 60 °C and increased denaturation temperature (89 °C) and enthalpy contribution of IgG and transferrin (see Table 1). The significant decrease in Δ*C_P_* compared to the controls is also a sign of structural rearrangement of serum proteins caused by the severity and poor outcomes of the aSAH. The biological variability in the DSC scans can also be clearly seen. Therefore, more samples are needed to verify the observations.

**Figure 4:**
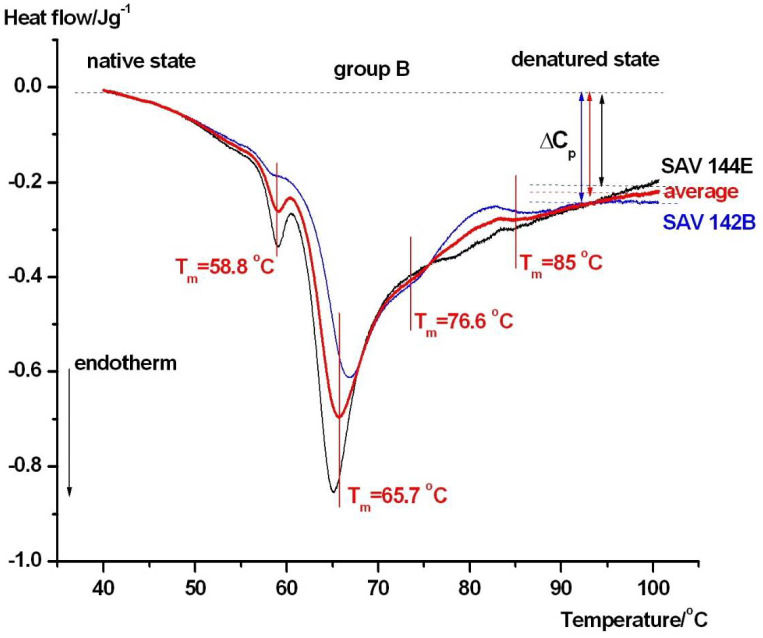
Thermal denaturation curves of patients in group B who were in a mild clinical state and had good outcomes. The denaturation peaks of possible thermal domains are marked on the average curve (red).

Group B had a median mRS score of 1, mild clinical state, and good outcomes. Their DSC scans can be seen in Figure 4.

The better clinical state and more favorable outcomes are clearly demonstrated by the DSc curves. The scans were similar to those of the controls: the albumin denaturation temperature and calorimetric enthalpy as well as those of IgG and transferrin are in nearly the same range as those of the controls, which clearly differs from the severely affected group (see Table 1).

**Figure 5:**
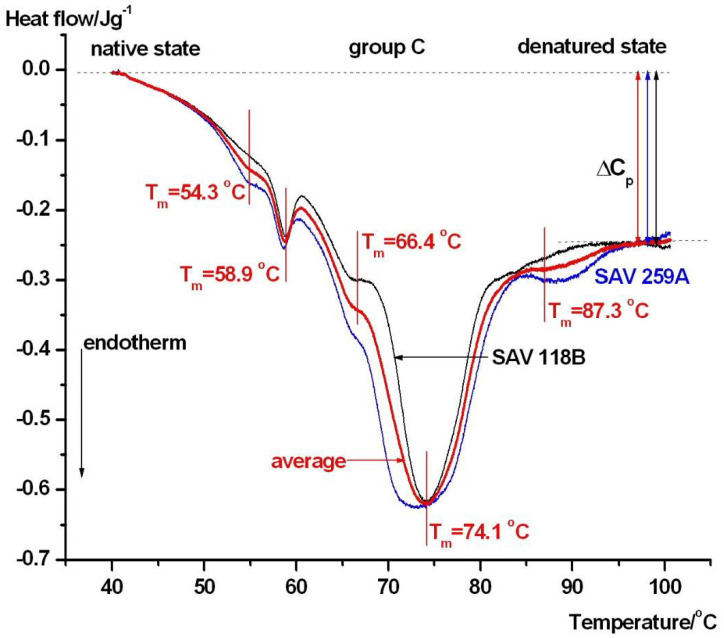
Thermal denaturation curves of patients in group C who were in a severe clinical state but with good outcomes. The denaturation peaks of possible thermal domains are marked on the average curve (red).

The DSC scans from **group C** who were in a severe clinical state but had good outcomes can be found in Figure 5.

The shape of the scans significantly differs from those of the control and **groups A** and **B**. The thermal stability profile of albumin in these scans exhibits three distinct thermal domains (with Tm values of approximately 59, 66.4, and 74 °C) and shows an increased calorimetric enthalpy that remains within the range of control values. (see Table 1). These findings indicate pronounced albumin thermal heterogeneity in this small subgroup; however, given the limited number of patients, this pattern cannot be considered a definitive phenotype. Comparison of the half-width (T_1/2_, defined as the temperature range at half of the maximum heat flow) of the albumin contribution in scans from control and different clinical-state groups reveals lower structural cooperativity of albumin in the clinical groups. (see Figures).

## 4. Discussion

DSC provides an integrative biophysical fingerprint of the serum proteome, which is dominated by albumin and immunoglobulins, while being highly sensitive to ligand binding, post-translational modifications, protein–protein interactions, and disease-related changes in protein composition. The serum component assignments in serum/plasma DSC scans are based on commonly used heuristic associations in the literature and should be interpreted as approximations because DSC provides an ensemble thermodynamic signal rather than protein-specific identification. Previous studies have established that pathological conditions induce reproducible alterations in serum DSC thermograms, which reflect systemic inflammatory and metabolic disturbances rather than isolated protein changes [19,20,21]. In the present study, distinct DSC profiles were observed between control subjects and the three clinical groups, indicating that early (day 1) serum protein thermal behavior reflects both disease severity and clinical outcome. In healthy controls (Figure 1 and Figure 2), the serum DSC profiles were characterized by a dominant albumin-related endothermic transition around 65–66 °C, accompanied by smaller higher-temperature components that were mainly attributed to immuno-globulins and transferrin. This pattern is consistent with previously reported reference thermograms of normal human sera, which reflects a largely homogeneous and cooperative denaturation of albumin under physiological conditions [21].

In contrast, the patients with severe clinical conditions and unfavorable outcomes (**Group A**, Figure 3) exhibited a pronounced reorganization of the DSC profile. The albumin-associated transition showed an upward shift in melting temperature (*T_m_*), accompanied by a reduction in albumin-related calorimetric enthalpy and a decrease in Δ*C_p_*. Simultaneously, the higher-temperature transitions, attributed to immunoglobulins and transferrin, displayed increased *T_m_* values and a greater relative contribution to the total heat capacity curve. Together, these changes indicate that although albumin appears thermally stabilized, its unfolding becomes less cooperative and energetically less favorable. Ligand-induced stabilization of albumin is a well-documented phenomenon. Albumin binds a wide range of endogenous and exogenous ligands, including fatty acids, metabolites, and drugs, many of which accumulate during critical illnesses. Binding of such ligands has been shown to increase albumin *T_m_* while altering unfolding enthalpy and heat capacity, resulting in modified DSC profiles [19]. In addition, aSAH is associated with an obvious systemic inflammatory response, immune activation, and oxidative stress, all of which can modify serum protein structure and composition [24,32]. These processes promote albumin oxidation, carbonylation, and complex formation, leading to reduced unfolding cooperativity and apparent thermal stabilization. In aSAH, an additional and disease-defining source of ligand loading arises from erythrocyte lysis and the release of free hemoglobin and heme into the extracellular space. Free heme is known to bind serum albumin with high affinity, and ligand-induced thermal stabilization of heme-binding proteins has been demonstrated using differential scanning calorimetry [33]. Therefore, hemorrhage severity, erythrocyte breakdown, and systemic heme burden represent plausible contributors to the increased albumin melting temperature and reduced unfolding cooperativity observed in patients with severe clinical conditions. Although free hemoglobin and heme concentrations were not measured in the present pilot study and serum DSC cannot resolve individual ligand–protein interactions, these mechanisms provide a biologically relevant framework for interpreting the observed thermogram reorganization. Future studies combining DSC with quantitative markers of hemolysis and heme metabolism will be required to directly test this hypothesis. The enhanced contribution and stabilization of higher-temperature components in **Group A** further supports the presence of immune activation and acute-phase protein redistribution. Clinical and experimental studies have consistently shown that neuroinflammatory responses following aSAH extend beyond the central nervous system and manifest systemically, influencing circulating immunoglobulin levels and protein–protein interactions [24,32]. Such systemic changes are known to produce characteristic shifts in serum DSC thermograms in inflammatory and neurocritical conditions [21]. Recent proteomics studies on aSAH have identified inflammasome-related proteins, including caspase-1, ASC, and IL-1β, as key mediators of neuroinflammation and systemic inflammatory responses with prognostic relevance [8]. Although DSC cannot resolve individual proteins, inflammation-driven changes in protein abundance, activation state, and interaction networks are expected to indirectly influence serum DSC thermograms. Increased levels of activated inflammatory and acute-phase proteins, together with altered protein–protein interactions and ligand binding, can modify the overall heat capacity profile and the contribution of higher-temperature thermal transitions. In this framework, DSC reflects the integrated thermodynamic consequence of proteomic remodeling rather than specific molecular events and should be considered complementary to molecular proteomics, providing a system-level readout of early inflammatory alterations after aSAH. The patients with mild clinical conditions and favorable outcomes (**Group B**, Figure 4) displayed DSC thermograms closely resembling those of healthy controls. Albumin *T_m_*, enthalpy, and the relative contribution of higher-temperature components remained mostly within the control range, suggesting minimal early systemic perturbation of the serum proteome. This observation aligns with clinical evidence indicating that limited systemic inflammation and preserved albumin homeostasis are associated with improved neurological outcomes after aSAH [24,32]. A distinct pattern was observed in patients with severe clinical conditions but favorable outcomes (**Group C**, Figure 5). In this group, the albumin-related transition fragmented into multiple components, with peaks observed around approximately 59, 66.4, and ~74 °C. Such multi-domain behavior indicates pronounced albumin heterogeneity, reflecting the coexistence of albumin populations with different thermal stabilities at the early post-ictus stage. While these patterns were observed in patients with favorable outcomes, no longitudinal DSC measurements were performed; therefore, reversibility or normalization of the thermogram cannot be inferred from the thermodynamic evidence. Experimental DSC studies have demonstrated that albumin heterogeneity can arise from variable ligand loading, redox state alterations, and transient conformational rearrangements, even in the absence of irreversible protein damage [19].

Importantly, despite severe clinical presentation, Group C’s thermograms did not exhibit the dominant immunoglobulin-driven reorganization that was characteristic of Group A’s thermograms. This suggests that the observed heterogeneity is more likely related to dynamic and potentially reversible metabolic adaptations rather than sustained inflammatory proteomic remodeling. Neurocritical care studies using indirect calorimetry have highlighted substantial interindividual variability in energy expenditure and substrate utilization following acute brain injury, emphasizing the metabolic instability of this patient population [24]. Such metabolic variability may directly influence serum ligand composition and albumin binding states, thereby producing complex but non-pathological DSC profiles. Albumin thermal heterogeneity is a well-documented phenomenon and may arise from several mechanisms independent of disease outcome. Variable ligand loading, including differences in fatty acid binding, accumulation of endogenous metabolites, drug–protein interactions, and alterations in albumin redox state, are all known to significantly modify albumin melting temperature, unfolding cooperativity, and peak multiplicity in DSC measurements. Acute systemic inflammation and oxidative stress following aneurysmal subarachnoid hemorrhage may further enhance this heterogeneity. Therefore, the multi-domain albumin-related transitions observed in Group C’s thermograms should not be interpreted as a distinct thermogram phenotype but rather as reflecting dynamic and potentially reversible biophysical states of albumin in a subset of patients. It is important to emphasize that DSC provides an integrated thermodynamic fingerprint of the serum proteome rather than protein-specific unfolding events. In the context of aneurysmal subarachnoid hemorrhage, profound systemic changes occur in acute-phase proteins, immunoglobulins, complement components, and protein–protein and protein–ligand interactions, all of which may influence the observed thermograms. While albumin is the most abundant serum protein and strongly shapes the dominant mid-temperature transition, changes attributed to albumin-associated domains should be interpreted within the broader context of global proteomic remodeling rather than as isolated albumin structural alterations. Overall, these findings indicate that serum DSC thermograms encode clinically relevant information beyond disease severity alone. While Group A’s profiles reflect sustained systemic inflammation and unfavorable outcomes, Group B’s profiles indicate relative systemic stability, and Group C’s profiles reveal marked but potentially reversible protein heterogeneity compatible with recovery. These results support the use of DSC scans as a systems-level biomarker capable of capturing early pathophysiological trajectories following aSAH.

This study has several limitations that should be acknowledged. First, because differential scanning calorimetry provides an integrative, ensemble-level thermodynamic signal and does not resolve individual molecular contributors, the present study cannot attribute the observed thermogram changes to specific proteins, post-translational modifications, or ligand-binding events, and all interpretations should therefore be considered at the proteome level. In addition, the relatively small sample size of this pilot study—particularly for the outcome-based subgroups—limits the statistical power and precludes robust subgroup analyses, including the evaluation of potential sex-related differences in serum DSC profiles. Second, the serum DSC measurements were performed at a single early time point (day 1 after ictus). As inflammatory responses and acute-phase protein alterations following aneurysmal subarachnoid hemorrhage often peak several days after the initial event, the present data only capture early-phase proteomic changes. The lack of longitudinal DSC measurements precludes assessments of temporal dynamics, causality, and thermogram normalization during recovery. Third, given that Group C consisted of only two to three patients, the observed DSC patterns may reflect individual variability rather than a reproducible group-level characteristic and therefore require validation in larger cohorts with serial sampling. Finally, potential confounding effects of medications, nutritional status, and metabolic interventions could not be fully controlled and may have influenced serum protein–ligand interactions and, consequently, the observed DSC thermograms.

## 5. Conclusions

In conclusion, serum DSC measurements capture early, disease-related alterations in the systemic proteome following aSAH and discriminate between distinct clinical severity and outcome groups. The observed thermograms reflect integrated effects of inflammation, metabolic stress, and protein–ligand and protein–protein interactions rather than isolated molecular changes. Although preliminary, these findings support the potential of DSC as a systems-level biomarker for early risk stratification and trajectory assessment after aSAH. Validation in larger, longitudinal cohorts will be essential to establish its prognostic utility.

## Figures and Tables

**Table 1:** Melting temperatures (Tm_1_–Tm_6_) and calorimetric enthalpy (ΔH_cal) obtained from DSC analysis of plasma samples from control male and female subjects and patient groups A–C. Values are reported as mean ± SD. Dashes indicate transitions that were not detected.

Samples	*T_m_*_1_/°C	*T_m_*_2_/°C	*T_m_*_3_/°C	*T_m_*_4_/°C	*T_m_*_5_/°C	*T_m_*_6_/°C	Δ*H_cal_*/Jg^−1^
ctr. male	56.3 ± 0.4	60.4 ± 0.3	65.2 ± 0.3	~71 ± 0.3	81.7 ± 0.2	88.9 ± 0.5	1.51 ± 0.07
ctr. female	55.9 ± 0.3	57.3 ± 0.5	65.7 ± 0.2	~72 ± 0.4	81.4 ± 0.3	92.5 ± 0.4	1.35 ± 0.06
group A	54.5 ± 0.4	59.9 ± 0.4	66.4 ± 0.2	71.8 ± 0.3	-	89.1 ± 0.4	1.09 ± 0.05
group B	-	58.8 ± 0.3	65.7 ± 0.3	76.6 ± 0.5	-	85 ± 0.4	1.27 ± 0.06
group C	54.3 ± 0.4	58.9 ± 0.3	66.4 ± 0.3	74.1 ± 0.4	-	87.3 ± 0.05	1.41 ± 0.07

## Data Availability

The original contributions presented in the study are included in the article; further inquiries can be directed to the corresponding author.

## Abbreviations

The following abbreviations are used in this manuscript:

aSAH	aneurysmal subarachnoid hemorrhage
CRP	C-reactive protein
CSF	cerebrospinal fluid
CT	computed tomography
CTA	computed tomography angiography
DCI	delayed cerebral ischemia
DSA	digital subtraction angiography
DSC	differential scanning calorimetry
ΔCp	change in heat capacity
ΔHcal	calorimetric enthalpy
EVD	external ventricular drain
HSA	human serum albumin
IgA	immunoglobulin A
IgG	immunoglobulin G
IQR	interquartile range
mFisher	modified Fisher score
MR	magnetic resonance
mRS	modified Rankin Scale
SD	standard deviation
Tm	melting denaturation temperature
WFNS	World Federation of Neurological Societies
